# Askings and Answers

**Published:** 1884-09

**Authors:** 


(20.) A boy of fifteen has not yet erupted his cuspids. The other teeth are in position, and the temporary cuspids were shed some time since. There is an almost constant pain and uneasiness referable to their locality, and the prominence in the alveolus over their probable position is quite marked. There is abundance of room for them, but they seem unreasonably delayed, and their cusps have not yet appeared through the bone. What shall be done with the case? DENTIST.

(21.) Will some of your contributors kindly inform me what causes the disease known as "pyorrhœa alveolaris?" IOWA.

(22.) I have been told that there is a way of making new rubber stick to old plates in repairing cases, and I will consider it a great favor if some reader of your excellent journal will be good enough to explain the process of doing it. M. S.

(23.) Some dentists are exceedingly particular about having the gum around a tooth thoroughly lanced before extracting it, while others never think of doing it, claiming that there is no necessity for using a lancet in such cases. I would like to get the views of dentists who have had better opportunities for deciding this matter than myself. L. R.

(24.) What has become of the Chalfant case? I refer to the dentist who shot Josiah Bacon, in San Francisco. A. B. C.

(25.) I am threatened with prosecution under the Richmond Crown Patents. Is there any reason why I should pay a royalty, and can the patent be sustained? GOLD CROWN.

Write to Dr. W. H. Dwinelle, No. 15 West 34th St., New York.—EDITOR.

### Answers.

NEW JERSEY (14.) Put in some kind of temporary stopping properly medicated, and *wait*—wait if need be a month, and ten to one the mass will die and admit of removal without trouble.

G. B., (15.) Yes. Remove the serrations of the permanent teeth in the mouth of young people. Smooth up the notched edges with an emery-paper disk.

C. F. M., (17.) Not absolutely "perfect." Practically solid would be a more "perfect" expression. GARRETT NEWKIRK, Chicago.

In reply to C. F. M., in the Aug. No. of the "I. P.," permit a reader to state that many people have a peculiar way of expressing their views. They do not always mean *exactly* what they say; and moreover it has been whispered that some individuals exist who are prone to color their remarks with a thin coating of lovely-tinted exaggeration. H. M.

